# Ester-Based Electrolyte Mixtures for Graphene Supercapacitors: A Molecular Dynamics Investigation

**DOI:** 10.1021/acs.jpcb.5c06507

**Published:** 2025-12-23

**Authors:** Lucas de S. Silva, Guilherme Colherinhas

**Affiliations:** Instituto de Física, 67824 Universidade Federal de Goiás, 74690-900 Goiânia, GO, Brazil

## Abstract

The development of sustainable and high-performance electrolytes is essential for advancing next-generation supercapacitors. In this study, we employed classical molecular dynamics simulations to investigate graphene-based supercapacitors using hydrated ester-based ionic liquids, both in pure form and in mixtures. The models are based in 1-butyl-3-methylimidazolium ([bmim]) cation and acetate ([ace]), benzoate ([bnz]) and propanoate ([prop]) anions. Structural analyses revealed well-defined electric double layers (EDLs) characterized by charge alternation across sublayers and moderate overscreening. Electrostatic potential profiles, obtained from the one-dimensional Poisson equation and corrected by the point of zero charge (PZC), exhibited a nearly linear response with surface charge density, with potential drops (ΔΦ) ranging from ∼1.1 V for [bmim]­[ace] + [bmim]­[bnz] to over 2.1 V for [bmim]­[prop]. Capacitance values confirmed these trends: the highest total capacitances were observed for [bnz]-containing mixtures (up to 2.83 μF/cm^2^ for [bmim]­[ace] + [bmim]­[bnz]), while [bmim]­[prop] and [bmim]­[ace] showed the lowest (≈2.5 μF/cm^2^). Energy density calculations highlighted a contrasting behavior: [bmim]­[prop], despite its lower capacitance, reached the largest gravimetric (4.06 J/g) and volumetric (4.58 J/cm^3^) energy densities due to its higher total potential difference. However, when normalizing the comparison at a fixed potential difference of 2.5 V, [bnz]-containing electrolytes—particularly [bmim]­[ace] + [bmim]­[bnz]—achieved the best performance, with gravimetric and volumetric energy densities of 6.44 J/g and 7.37 J/cm^3^, respectively. These results emphasize the decisive role of the [bnz] anion in tuning interfacial structure and energy storage, providing valuable guidelines for the rational design of ester-based ionic liquid electrolytes for sustainable supercapacitors.

## Graphical abstract

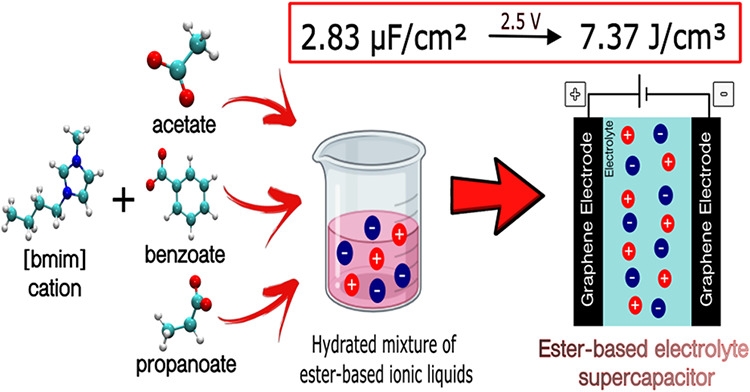

## 1 Introduction

The development of electrochemical energy storage devices (EESDs), such as rechargeable batteries and supercapacitors (SCs), has advanced considerably in response to the growing demand for solutions that combine high efficiency, fast response, and long-term cycling stability.^1–3^ Among these devices, SCs stand out by offering outstanding power density and extended lifespan, both crucial features for applications that demand dynamic performance and operational robustness.^4–6^ Esters are organic compounds formed by the reaction between acids (organic or inorganic) and alcohols, containing the characteristic −COO– functional group.^7^ They are polar molecules capable of accepting (but not donating) hydrogen bonds and interacting through dipole–dipole and van der Waals forces.^8,9^ Esters exhibit intermediate polarity, higher than ethers but lower than alcohols, which provides good solvation capacity and moderate volatility.^9^ Moreover, many esters, particularly short-chain ones, have relatively low viscosity, which favors molecular mobility.^10^

Previous studies have reported that their addition can reduce electrolyte viscosity and increase salt solubility (for example, tetraalkylammonium salts) thereby enhancing conductivity and overall electrochemical performance. Specifically, esters used as additives in ethylene carbonate (EC) have been shown to improve stability, conductivity, and energy storage capacity in supercapacitors.^11^ These effects arise not only from the intrinsic properties of esters but also from their collective behavior in mixtures. Mixed solvents are widely employed in electrolytes to tailor macroscopic properties such as dielectric constant, melting/boiling points, and viscosity.^12^ In our previous work,^13^ we demonstrated that mixtures of ionic liquids based on the 1-Butyl-3-methylimidazolium ([bmim]) cation with different anions (bromide, perchlorate, and nitrate), combined with water, represent a promising strategy for improving the performance of graphene-based supercapacitors. Furthermore, previous studies^14–22^ have demonstrated that electrolyte hydration can serve as a beneficial strategy to enhance the electrochemical performance of devices in this category.

Beyond the electrolyte optimization through hydrated ionic liquid mixtures,^23–26^ several electrode-oriented strategies have also been explored to enhance the performance of SCs. Several studies have demonstrated the remarkable performance of graphene electrodes: aminated reduced graphene oxide (NH_2_–rGO) produced via rapid, energy-efficient methods shows outstanding electrochemical stability;^27^ graphene/activated carbon hybrids exhibit low internal resistance and high durability;^28^ flexible reduced graphene oxide films incorporating lignosulfonate and carbon microspheres achieve capacitances as high as 178 F g^–1^, far surpassing pristine rGO.^29^ On the other hand, computational studies have reinforced these findings by showing how graphene’s structural and chemical features tune its electrochemical response.^23–26,30,31^

Despite these advances, individual studies addressing how mixtures of ionic liquids containing ester-derived anions specifically affect the electrode–electrolyte interface in supercapacitors remain scarce. Therefore, in this work, we employ classical molecular dynamics (MD) simulations to investigate how ester mixtures influence the structure of the electric double layer (EDL), the differential potential, capacitance, and ion transport in graphene-based systems. More specifically, we focus on seven distinct cosolvated models based on acetate ([ace]), benzoate ([bnz]) and propanoate ([prop]) with water, namely: (M_A_) [bmim]­[ace] + *H*_2_ *O*; (M_B_) [bmim]­[bnz] + *H*_2_ *O*; (M_C_) [bmim]­[prop] + *H*_2_ *O*; (M_AB_) [bmim]­[ace] + [bmim]­[bnz] + *H*_2_ *O*; (M_AC_) [bmim]­[ace] + [bmim]­[prop] + *H*_2_ *O*; (M_BC_) [bmim]­[bnz] + [bmim]­[prop] + *H*_2_ *O*; and (M_ABC_) [bmim]­[ace] + [bmim]­[bnz] + [bmim]­[prop] + *H*_2_ *O*.

## 2 Computational Details

Classical MD simulations were conducted to explore the structural and electrostatic characteristics of seven graphene-based supercapacitor systems containing ester-based electrolyte mixtures. The investigated samples were prepared from different combinations of the ionic liquid 1-butyl-3-methylimidazolium ([bmim]) with distinct anions, always in the presence of water. They were designated as follows:

- M_A_–system composed of [bmim]­[ace] (1-butyl-3-methylimidazolium acetate) in aqueous solution;
- M_B_–system consisting of [bmim]­[bnz] (1-butyl-3-methylimidazolium benzoate) in aqueous solution;
- M_C_–system prepared with [bmim]­[prop] (1-butyl-3-methylimidazolium propionate) in aqueous solution;
- M_AB_–binary mixture containing both [bmim]­[ace] and [bmim]­[bnz] in aqueous solution;
- M_AC_–binary mixture of [bmim]­[ace] and [bmim]­[prop] in aqueous solution;
- M_BC_–binary mixture of [bmim]­[bnz] and [bmim]­[prop] in aqueous solution;
- M_ABC_–ternary system comprising [bmim]­[ace], [bmim]­[bnz], and [bmim]­[prop], in aqueous solution.

The electrolyte concentration was kept constant in 2 M for all models. The number of ionic liquid ion pairs in each simulation cell was computed directly from the molar concentration relation *C* = *n*/*V*, and, in the case of mixed electrolytes, this total was apportioned among species according to the target composition. Water molecules were added without altering the total ionic concentration, ensuring that all systems represent a 2 M electrolyte environment. To avoid arbitrary parametrization and guarantee thermodynamic consistency, the number of molecules for each species was obtained analytically by combining the concentration definition, density–mass relations, and Avogadro’s law, assuming approximate additivity of volumes. The resulting closed-form equations for systems containing one, two, and three ionic liquids plus water are provided in the Supporting Material. The composition information about the systems is highlighted in Table 1. Two simulation stages were performed: an initial ∼15 ns run to achieve thermodynamic equilibration, followed by extended ∼50 ns production runs dedicated to analyzing the structural organization and electrostatic behavior of the supercapacitors. The simulations were carried out at 500 K, with temperature control achieved via the v-rescale thermostat^32^ using a coupling constant of 0.1 ps. It is important to note that all simulations were carried out in the NVT ensemble. Although this temperature exceeds typical operating conditions of supercapacitors, it is a well-established strategy in molecular-dynamics simulations of ionic-liquid electrolytes to enhance sampling efficiency and reduce correlation between configurations in highly viscous systems. Working in NVT at elevated temperature accelerates ion mobility and improves the ergodicity of the ensemble without modifying the electrode charge distribution or the qualitative nature of the electric-double-layer structure. Therefore, the use of 500 K ensures statistically robust sampling and does not affect the physical interpretation of the interfacial phenomena investigated here. It is important to highlight to enhance sampling efficiency in viscous ionic systems, simulations were carried out at 500 K, a temperature commonly used in classical MD studies of ionic liquids and carbon–based supercapacitors to accelerate decorrelation without altering the imposed electrode charge distribution. While increasing the temperature affects dynamical time scales quantitatively, previous studies have shown that the qualitative structural features of the electric double layer—such as ion layering, preferential adsorption, and relative interfacial affinities—are largely preserved across a wide temperature range. Thus, the trends reported here reflect robust structural behavior rather than temperature-specific dynamics.^23–26,33,34^ Long-range electrostatics were treated with the Particle–Mesh Ewald (PME) method^35^ and a real-space cutoff of 1.3 nm, while van der Waals forces were modeled with a Verlet cutoff scheme, also employing a 1.3 nm cutoff. The simulation cells were prepared using the PACKMOL package,^36^ where molecular species were randomly placed to reproduce the target density, adjusted by tuning the *z*-axis spacing between the electrodes. The graphene electrodes measured 3.705 × 3.853 nm^2^ and were separated by approximately 12 nm, providing the region in which the electrolytes were confined. To avoid spurious periodic interactions, a vacuum slab of 24 nm was inserted along the *z*-direction. Representative molecular structures of the electrolyte components, the full box simulation and the corresponding model *M*_ABC_ supercapacitor cell is depicted in Figure 1.

**Table 1:** System Compositions Analyzed in This Work^a^

model	#[bmim]	#[ace]	#[bnz]	#[prop]	#H_2_O	*m*_Tot_ (× 10^–19^ kg)	#atoms	#H_2_O/#IL
M_A_	149	149	--	--	4.135	1.94	18.253	28
M_B_	139		139	--	3.857	1.97	18.072	28
M_C_	145		--	145	4.024	1.93	18.227	28
M_AB_	144	72	72	--	4.016	1.96	18.240	28
M_AC_	146	73	--	73	4.094	1.94	18.253	28
M_BC_	142	--	71	71	3.958	1.96	18.208	28
M_ABC_	144	48	48	48	4.022	1.95	18.234	28

a The table summarizes, for each system, the number of ionic liquid species, the number of water molecules, the total system mass (including both electrolyte and electrodes), and the total atom count (electrolyte plus electrodes). In addition, it reports the ratio of water molecules to [bmim] cations. In all cases, the electrolyte concentration was maintained at approximately 2 M.

**Figure 1:**
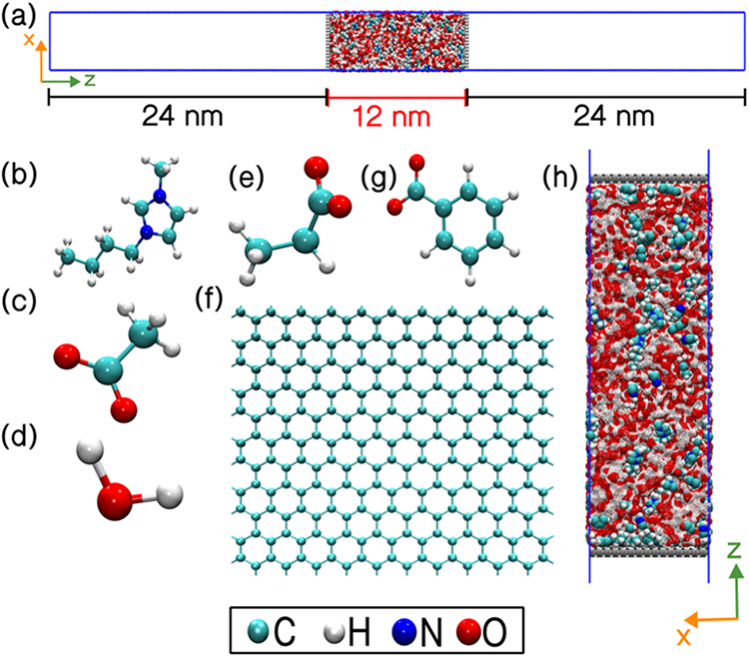
Panel (a) shows a representative snapshot of the full simulation box. Panels (b–g) depict the individual molecular components of the electrolyte: (b) [bmim]; (c) [ace]; (d) H_2_O; (e) [prop]; (f) graphene; and (g) [bnz]. Panel (h) illustrates the M_ABC_ supercapacitor system.

The force field parameters employed to describe each ionic liquid (IL) molecule were adapted from OPLS-AA,^37–40^ whereas water molecules were represented using the TIP3P model.^41^ For every model, four simulations were carried out with different surface charge densities (σ) applied directly to the electrodes: ±0.00, ±1.60, ±3.20, and ±4.81 μC·cm^–2^. These charges were uniformly distributed over the carbon atoms of the graphene electrodes (540 atoms per electrode), corresponding to atomic charges of ±0.00000000*e*, ±0.00264566*e*, ±0.00529132*e*, and ±0.00793697*e* per carbon atom, respectively for each σ values. This procedure mimics the progressive charging process of the supercapacitors. A constant charge (CCM) model was employed, in which partial charges remain fixed on the electrode atoms. This method has been demonstrated to provide reliable results within this voltage range, as it avoids the artificial polarization artifacts that may arise above ∼2 V, while still capturing realistic electrochemical behavior in good agreement with experimental observations.^42^ Moreover, it offers a substantially lower computational cost compared to more demanding approaches such as the constant potential method following the methodology used in previous studies.^14,43–45^ The constant-charge approach was adopted to model graphene electrodes. Although constant-potential models offer a more detailed treatment of electrode polarization, constant-charge simulations remain an established methodology for ionic-liquid-based supercapacitors and enable direct comparison among different electrolyte compositions under identical interfacial charge conditions. This strategy avoids the introduction of additional fitting parameters related to electrode charge equilibration and provides reliable insight into the relative structuring, adsorption behavior, and interfacial dynamics induced by changes in electrolyte composition and hydration level. Therefore, the use of the constant-charge model ensures computational consistency and accurate mechanistic interpretation of the electrolyte response to a defined electrode polarization environment. Bond constraints were treated with the LINCS (Linear Constraint Solver) algorithm,^46^ and all molecular dynamics simulations were performed using the GROMACS 2023 (Groningen Machine for Chemical Simulation) package.^47^

On the other hand, to evaluate the electrical properties of each system, the one-dimensional Poisson equation was employed to describe the potential profile along the *z*-axis, as expressed in $\Phi \left(Z\right)=-\frac{1}{\epsilon }\int_{-Z_{0}}^{Z}(Z-z^{\prime })\rho_{z}\left(z^{\prime }\right)dz^{\prime }$ .^23–26^ From the potential values at the electrodes, the total potential difference, corrected by the potential of zero charge (PZC),^23–26^ was determined for each device as follows: ΔΦ = δΦ^+^ – δΦ^–^, where δΦ ^±^ = Φ^+^ – Φ^–^. A subsequent linear fit of the form φ­(*x*) = α*x* + β was then performed, enabling the construction of the σ × Φ plot, where the slope of the fitted line corresponds to the specific capacitance of each electrode. Since the electrodes are connected in series, the total capacitance was obtained using the relation: $\frac{1}{C^{\mathrm{Tot}}}=\frac{1}{C^{+}}+\frac{1}{C^{-}}$ . Finally, the gravimetric and volumetric energy storage densities were also evaluated through the corresponding equations $u_{m}=\frac{1}{2m}C^{\mathrm{Tot}}{\left(\Delta \Phi \right)}^{2}$ and $u_{v}=\frac{1}{2v}C^{\mathrm{Tot}}{\left(\Delta \Phi \right)}^{2}$ , where *m* is the total mass and *v* is the volume of the supercapacitor.

## 3 Results and Discussion

### 3.1 EDL Structural Analysis

In SCs, the interfacial region adjacent to the electrodes plays a decisive role in determining their electrical properties. In simulations employing the constant-charge method, the fixed charges assigned to the electrode atoms give rise to an interfacial electrostatic environment that drives the reorganization of the electrolyte ions. Positively charged species tend to accumulate in the vicinity of the negatively polarized electrode, whereas negatively charged species approach the positively polarized electrode. This process gives rise to the electric double layer (EDL), which can be formally described in terms of distinct substructures. The innermost region, known as the Stern layer, consists of specifically adsorbed counterions that are strongly bound to the electrode surface, often involving short-range interactions such as van der Waals forces or partial charge transfer. Immediately beyond this compact layer lies the diffuse layer, where the remaining counterions are distributed according to a balance between electrostatic attraction and thermal motion, leading to a gradual decay of the potential into the bulk electrolyte. Together, these regions form the classical Stern–Gouy–Chapman model of the EDL, which provides a structural framework for understanding charge storage at the electrode–electrolyte interface. Figure 2 displays the mass density profiles of the electrolyte components in the vicinity of the positive electrode, whereas Figure 3 presents the corresponding mass density profiles near the negative electrode. Both Figures considering a surface density charge of ±4.81 μC/cm^2^ in the electrodes.

**Figure 2:**
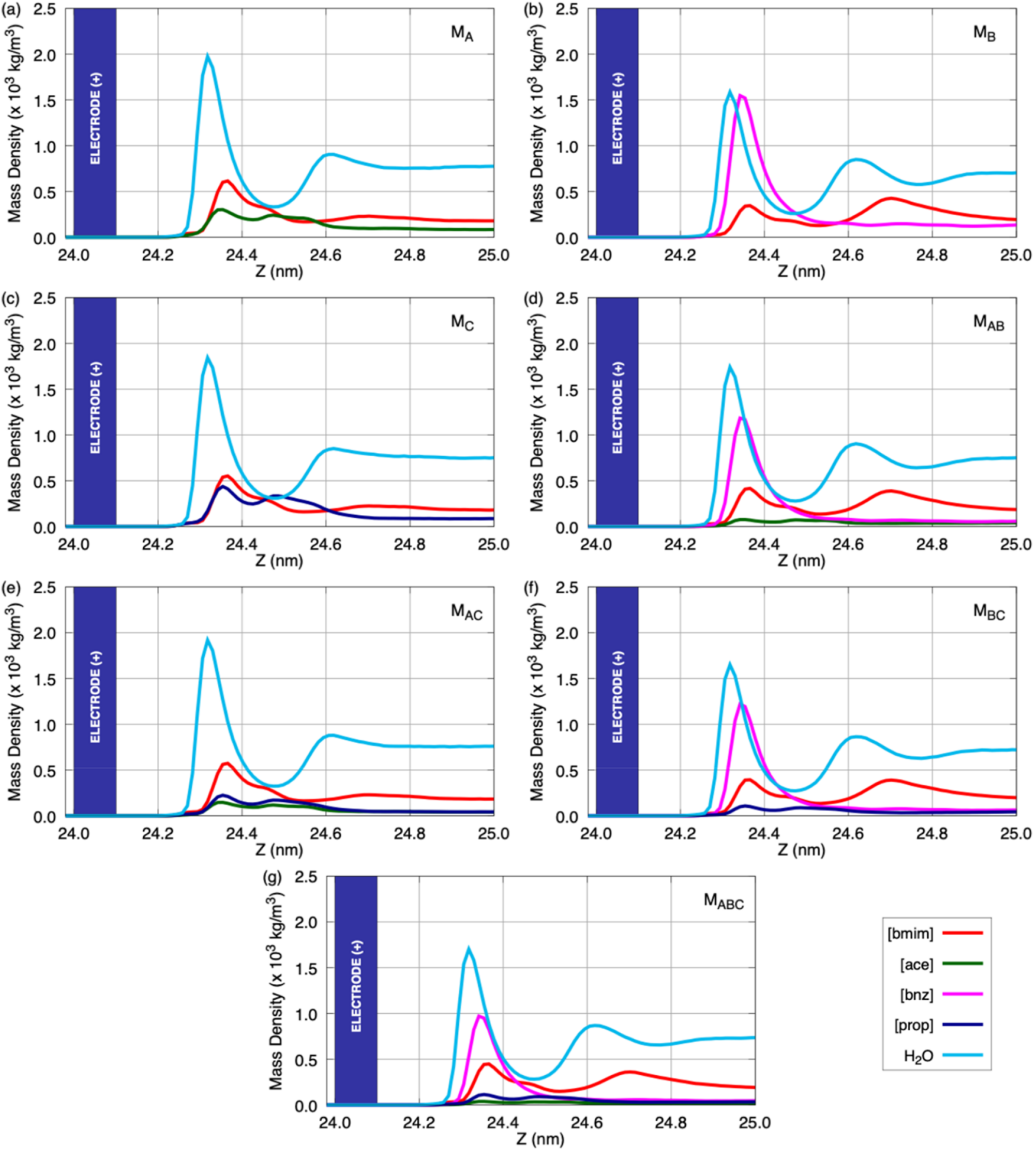
Mass density profiles of the electrolyte species in the vicinity of the positive electrode for the following models: (a) M_A_; (b) M_B_; (c) M_C_; (d) M_AB_; (e) M_AC_; (f) M_BC_; and (g) M_ABC_. The blue bar indicates the position of the positive graphene electrode.

**Figure 3:**
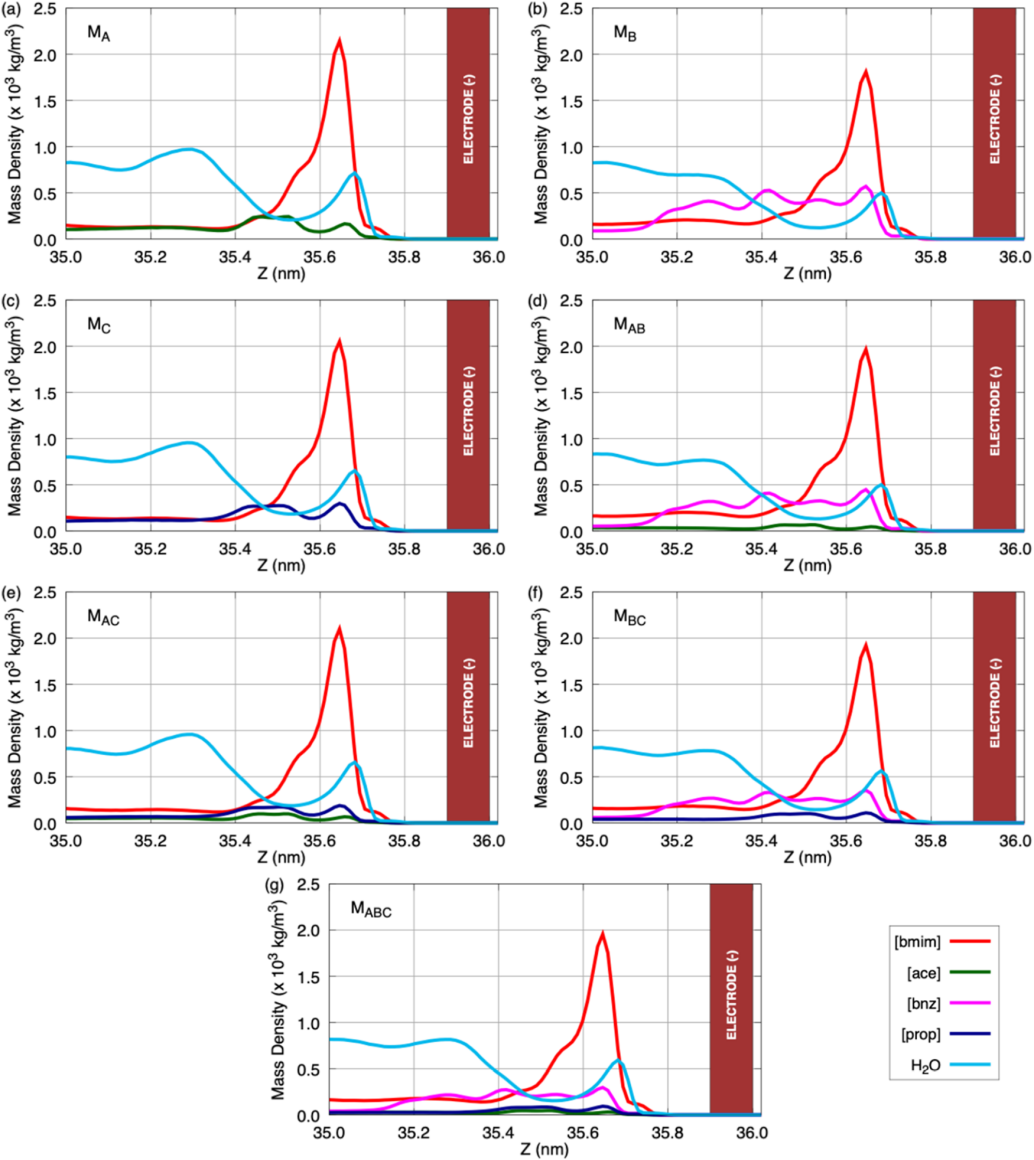
Mass density profiles of the electrolyte species in the vicinity of the negative electrode for the following models: (a) M_A_; (b) M_B_; (c) M_C_; (d) M_AB_; (e) M_AC_; (f) M_BC_; and (g) M_ABC_. The red bar indicates the position of the negative graphene electrode.

As shown in Figure 2, all models display a pronounced accumulation of water molecules near the positive electrode, with significant peaks located at approximately ∼0.3 nm from the electrode surface. This behavior reveals a marked infiltration of water near the positively charged electrode and reflects a highly organized interfacial structure primarily governed by the strong dipolar nature of water molecules. Under the applied electric field, the oxygen atom–bearing a partial negative charge–tends to orient toward the positively polarized graphene surface, promoting preferential adsorption in this region. Due to its small molecular size and high polarity, water also effectively competes with anions for interfacial occupancy, allowing it to penetrate the first adsorption layer more readily than bulkier organic ions. Additionally, the presence of the charged surface induces a partial disruption and subsequent rearrangement of the hydrogen-bond network, leading to the formation of a well-defined and densely packed hydration layer adjacent to the electrode. Regarding the ionic species, the M_A_ model ([bmim]­[ace] + *H*_2_ *O*) exhibits a rather peculiar behavior: the [ace] anion shows the lowest peak among all species at the positive electrode, whereas a higher accumulation of this ion—followed by [bmim] cations—would be expected. A similar trend is observed in the M_C_ ([bmim]­[prop] + *H*_2_ *O*) and M_AC_ ([bmim]­[ace] + [bmim]­[prop] + *H*_2_ *O*) models. Conversely, the behavior of the ionic species in M_B_, M_AB_, M_BC_, and M_ABC_ aligns more closely with the expected trend, as the [bnz] anion exhibits the most pronounced peaks in the vicinity of the positive electrode. In the M_B_ model ([bmim]­[bnz] + *H*_2_ *O*), the peak magnitude of [bnz] is nearly comparable to that of water molecules. For the mixed systems containing [bnz], this anion appears to play a dominant role in shaping the EDL, since the peaks of the other anions remain relatively small and consistently lower than those of the [bmim] cation.

In contrast, the behavior near the negative electrode follows the expected trend: water infiltration remains limited across all models, while [bmim] consistently dominates the first interfacial layers. Similar to the positive electrode, acetate and propionate contribute only weakly to the structuring of the EDL. Whenever benzoate is present, however, it appears more frequently in the interfacial region, in agreement with the species-counting results (Table 2) and with the stronger benzoate–electrode interaction energies obtained from the Coulomb and Lennard-Jones analyses (Figures 5 and 6). These energetic descriptors provide a direct microscopic explanation for the higher interfacial presence of benzoate compared to acetate and propionate, without requiring assumptions regarding hydration-shell strength or intrinsic affinity trends. Thus, the enhanced accumulation of benzoate results naturally from its larger ion–electrode interaction energies and from the more effective local charge compensation observed in the integrated charge profiles, reinforcing its leading role in defining the structure of both EDLs.

**Table 2:** Number of Species for Model Considering Three Different Regions Extending ∼1 nm from Each Electrode of the Supercapacitor: EDL+ (for positive electrode), Bulk (supercapacitor's central region) and EDL– (for negative electrode). Considering 10,000 Configurations from Molecular Dynamics Trajectory

model	specie	EDL+	bulk	EDL–
M_A_	[bmim]	11	120	18
	[ace]	15	120	14
	*H*_2_ *O*	274	3645	216
M_B_	[bmim]	11	110	18
	[bnz]	16	107	16
	*H*_2_ *O*	231	3459	168
M_C_	[bmim]	10	117	18
	[prop]	15	116	14
	*H*_2_ *O*	264	3553	207
M_AB_	[bmim]	11	114	19
	[ace]	5	64	4
	[bnz]	10	49	12
	*H*_2_ *O*	249	3591	176
M_AC_	[bmim]	10	117	18
	[ace]	7	60	6
	[prop]	8	57	8
	*H*_2_ *O*	269	3616	208
M_BC_	[bmim]	11	113	18
	[bnz]	11	50	10
	[prop]	4	62	5
	*H*_2_ *O*	241	3535	182
M_ABC_	[bmim]	11	115	18
	[ace]	2	43	3
	[bnz]	8	31	8
	[prop]	4	40	4
	*H*_2_ *O*	247	3587	188

Based on these rather peculiar behaviors, we sought to investigate in greater detail the formation of the EDLs. To this end, 10^4^ configurations were extracted from the MD-trajectory, from which species counting and hydrogen bond (HB) analysis were performed within a region extending ∼1 nm from each electrode (considering ±4.81 μC/cm^2^ as a surface density charge). It is worth emphasizing that, since the electrolytes are composed of ester-based ionic liquids, HBs plays a crucial role in the structuring of the EDLs for protic molecules. This is because ester species are unable to form HBs among themselves, as they cannot act as proton (*H* ^+^) donors, thereby restricting such interactions primarily to water molecules and other suitable partners. Table 2 shows the number of species in in the three distinct regions of the supercapacitors, namely the positive EDL (EDL+, for positive electrode), the bulk (central region), and the negative EDL (EDL−, for negative electrode), while as illustrated in Figure 4, the normalized number of HBs per water molecules was evaluated in the same regions is highlighted.

**Figure 4:**
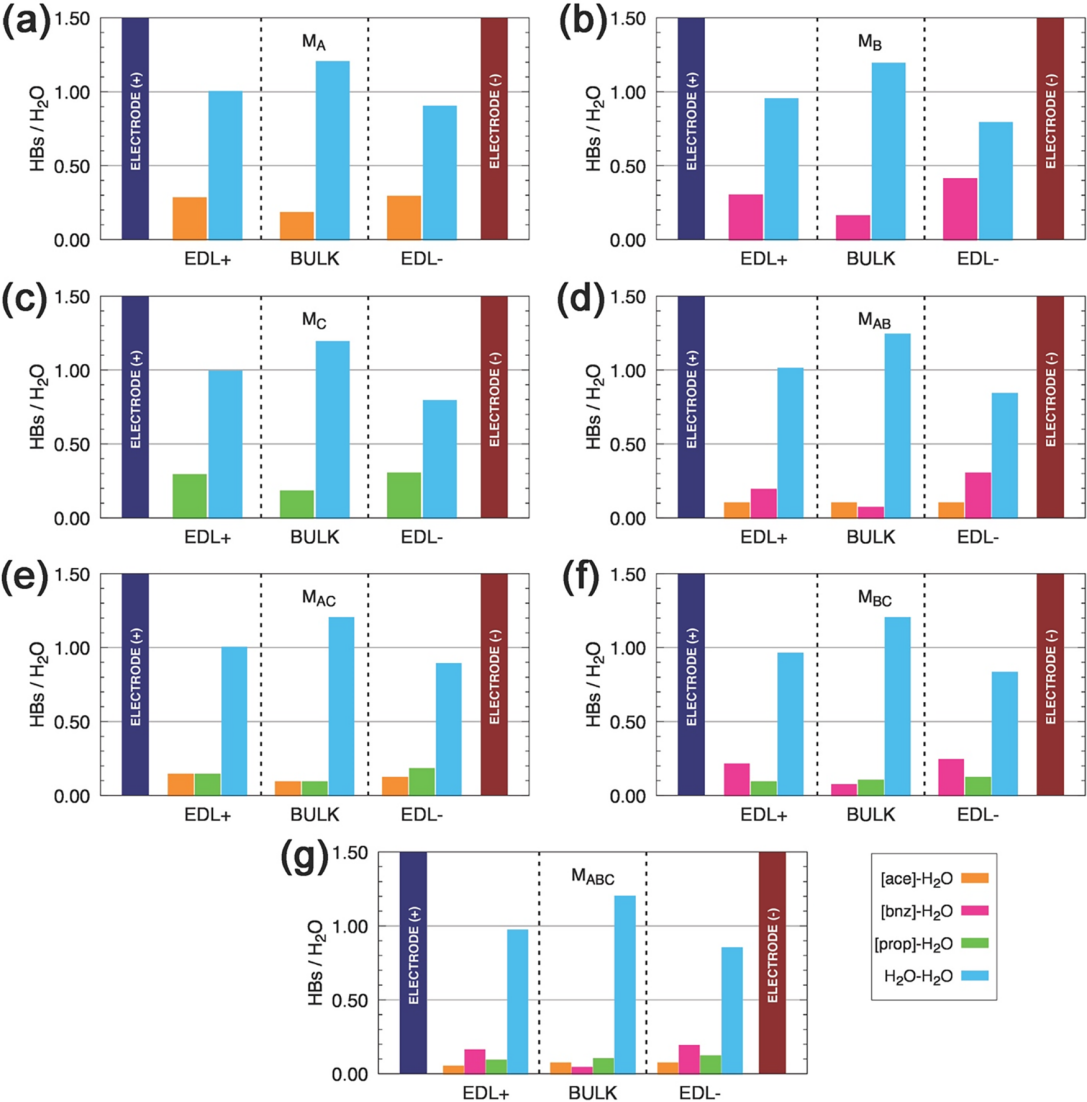
Normalized number of hydrogen bonds (HBs) per water molecules in the three regions of the supercapacitors: positive EDL (EDL+, for positive electrode), bulk (supercapacitor's central region), and negative EDL (EDL−, for negative electrode) for the following models: (a) M_A_; (b) M_B_; (c) M_C_; (d) M_AB_; (e) M_AC_; (f) M_BC_; and (g) M_ABC_.

For all models, a high concentration of water molecules is clearly observed in the positive EDL, consistent with the trends identified in the mass density profiles. Overall, in this region, the ratio between anions and cations remains nearly constant at approximately 15 negative ions to 11 positive ones, which is remarkable given that the global net charge is effectively conserved across all systems in this region. However, for the mixed systems (M_AB_, M_AC_, M_BC_, and M_ABC_), the relative proportions of each anion must be analyzed individually. A notable feature is the dominant behavior of the [bnz] anion, which consistently appears in larger amounts compared to the other species. For instance, in the M_AB_ system ([bmim]­[ace] + [bmim]­[bnz]), the ratio is about 2:1, with [bnz] present in twice the amount of [ace] in the positive EDL. In the M_BC_ system ([bmim]­[bnz] + [bmim]­[prop]), this ratio increases to approximately 2.75 [bnz] ions per [prop]. In the ternary M_ABC_ model, [bnz] again predominates, with 8 species detected, compared to only 2 [ace] and 4 [prop]. These findings strongly suggest that [bnz] anions play a leading role in shaping the positive EDL whenever they are present in the electrolyte, exerting a stronger influence than the other negative species considered in this study.

In the central region of supercapacitors (bulk region), the number of positive and negative ions is nearly equivalent, maintaining a ratio close to 1:1. Indeed, water molecules are considerably more abundant in this region compared to the other species. Near the negative electrode, a behavior like that at the positive electrode is observed, with a pronounced accumulation of water molecules. However, in terms of ionic distribution, [bmim] cations dominate, particularly at ∼0.6 nm from the electrode surface (as evidenced in the mass density profiles). Overall, the ratio of anions to cations remains nearly constant, at approximately 15 negative ions to 18 positive ones. For the mixed systems, the [bnz] anion again emerges as the most influential species. In the M_AB_ model, the ratio is about 3:1 ([bnz]:[ace]); in the M_BC_ model, it is approximately 2:1 ([bnz]:[prop]); and in the ternary M_ABC_ model, the ratio is close to 2:1:1 ([bnz]:[ace]:[prop]). Therefore, these results indicate that, like the positive EDL, the [bnz] anion plays a decisive role in shaping the structure of both EDLs, highlighting its strong interfacial influence and potential relevance to the electrochemical performance of the SCs.

The analysis of #HBs normalized per water molecule (#HBs/#H_2_O), as shown in Figure 4, provides crucial insights into the electrolyte structuring in the different models. In the bulk region, the water–water HB network reaches its maximum in all systems, reflecting the strong connectivity of water in a relatively homogeneous environment, far from the direct influence of the electrode fields. This connectivity ensures ion mobility and efficient mass transport, functioning as a reservoir of HBs. In the positive EDL, however, a clear suppression of water–water connectivity is observed, accompanied by an enhancement of anion–water HBs. This effect is particularly pronounced in systems containing [bnz], which show a larger number of [bnz]–water hydrogen bonds in the interfacial region. This reflects the different participation of each anion in the local HB network, but does not imply an independent quantification of interfacial affinity. Instead, the structural role of each anion at the interface is later confirmed by their computed ion–electrode interaction energies (Figures 5 and 6). In contrast, in the M_A_, M_C_, and M_AC_ models, composed of [ace] and/or [prop], the water–anion interactions are weaker, and the water–water network retains relatively higher connectivity.

**Figure 5:**
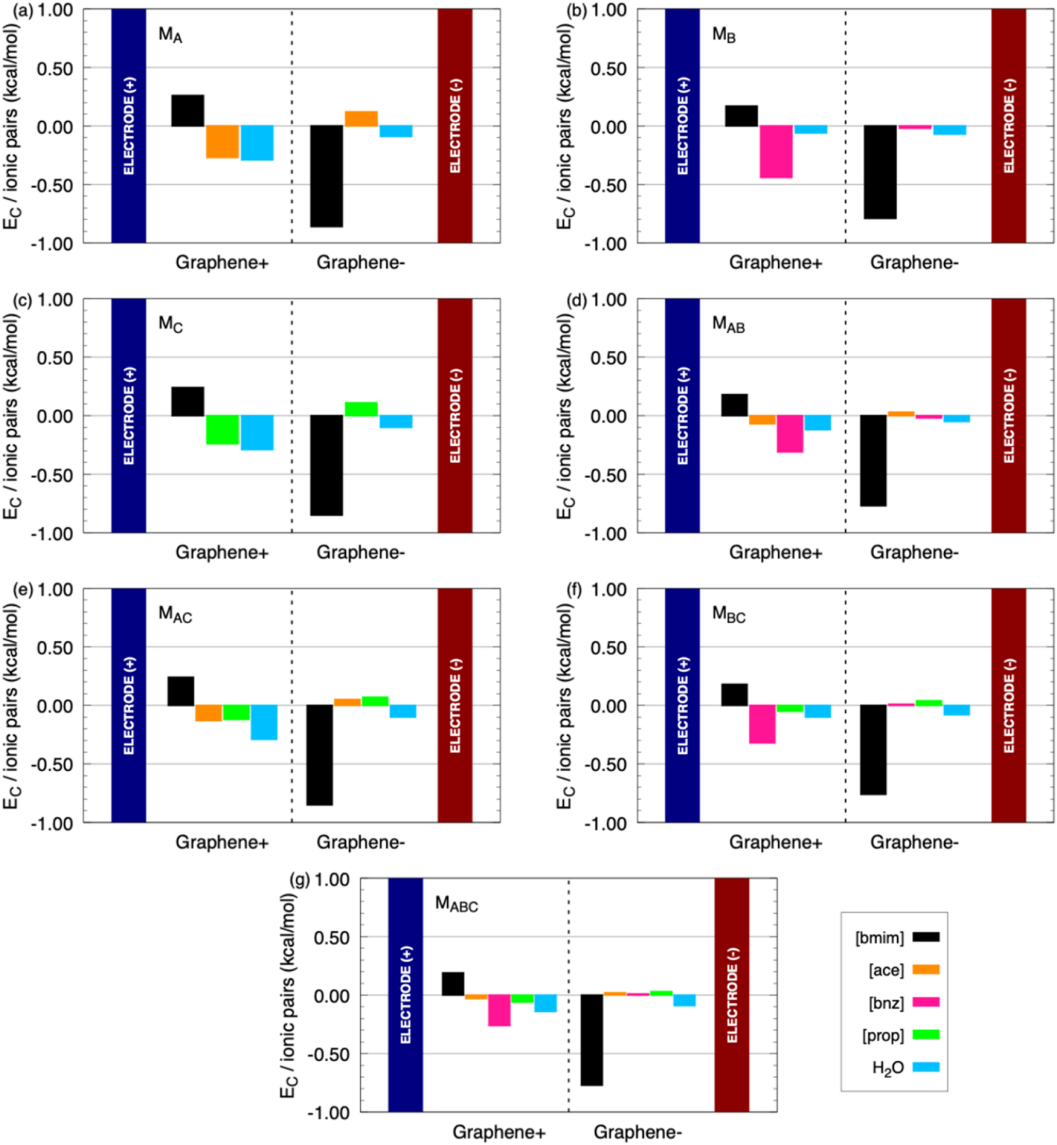
Coulomb energy interactions (*E*_C_) per ionic pairs considering a surface density charge of ±4.81 μC/cm^2^ in both electrodes for the following models: (a) M_A_; (b) M_B_; (c) M_C_; (d) M_AB_; (e) M_AC_; (f) M_BC_; and (g) M_ABC_.

**Figure 6:**
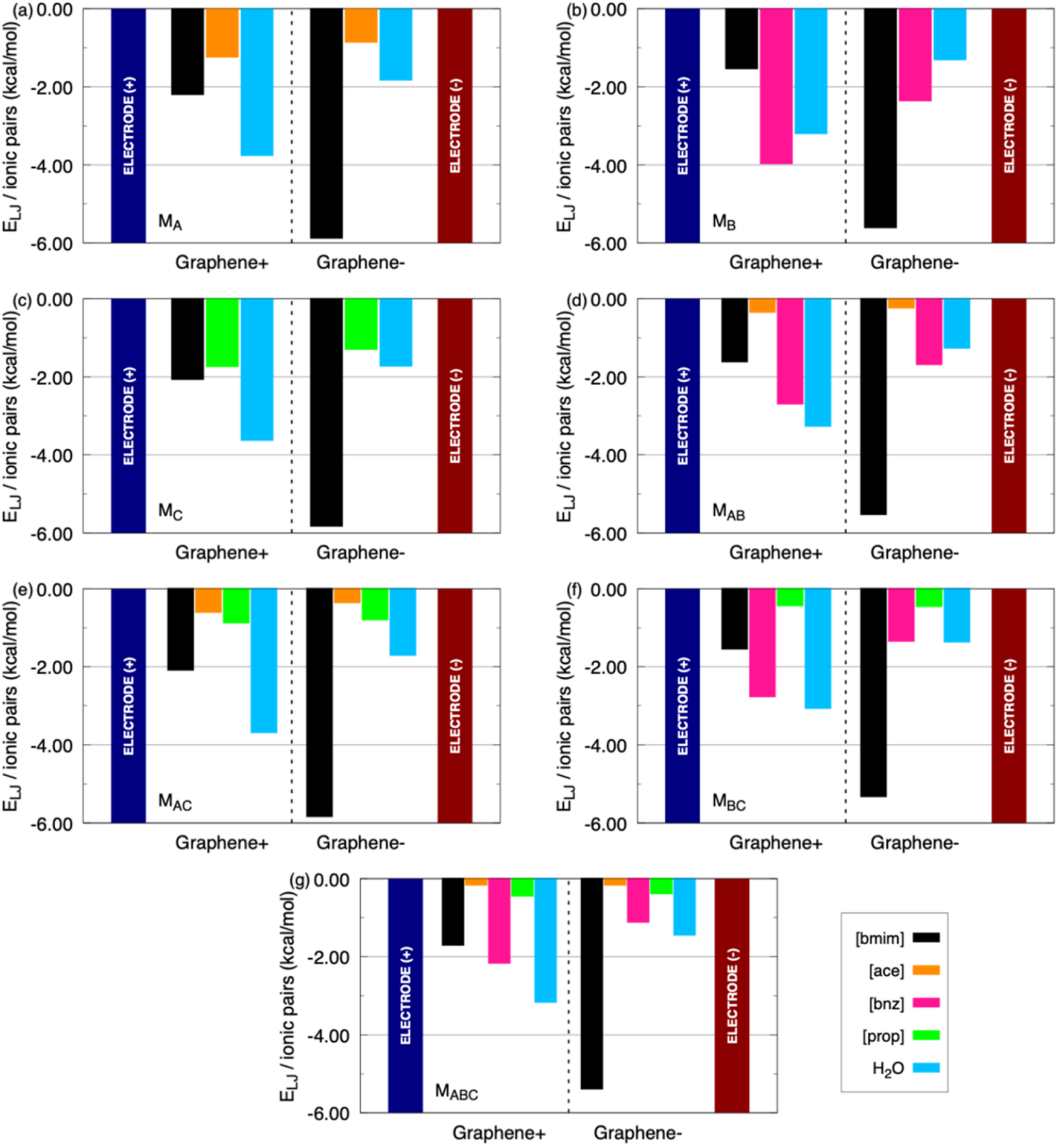
Lennard-Jones energy interactions (*E*_LJ_) per ionic pairs considering a surface density charge of ±4.81 μC/cm^2^ in both electrodes for the following models: (a) M_A_; (b) M_B_; (c) M_C_; (d) M_AB_; (e) M_AC_; (f) M_BC_; and (g) M_ABC_.

In the EDL–, the trend differs: the accumulation of [bmim] cations near the electrode significantly reduces the contribution of anion–water HBs. As a result, water–water interactions remain dominant but are less pronounced than in the bulk, suggesting that the structuring of the negative EDL is governed more by water redistribution and cationic influence than by anion-mediated HB. Overall, the results demonstrate that the [bnz] anion plays a key role in shaping the positive EDL, establishing stronger interactions with water and directly influencing the potential profile and differential capacitance. In mixed systems (M_AB_, M_BC_, and M_ABC_), [bnz] consistently dominates over [ace] and [prop], confirming its stronger interfacial role and suggesting that its presence contributes to enhanced structuring of the EDL and, consequently, to superior electrochemical performance.

Still in the framework of EDL formation, the Coulomb interaction energy (*E*_C_) and the Lennard-Jones interaction energy (*E*_LJ_) between all species and the electrodes were evaluated. Our analysis focuses on ion–electrode interactions and EDL organization, as these features govern charge compensation and capacitive behavior in constant-charge supercapacitor models. Although no explicit energetic decomposition among ions in solution was performed, ion–ion and ion–water interactions are inherently captured by the electrostatic and Lennard-Jones potentials in the force field, which dictate solvation, screening, hydrogen bonding, and competitive adsorption. The observed trends in mixed-anion systems therefore arise from the natural interplay among these interactions, rather than from the absence of them. This approach isolates the electrode-driven mechanisms relevant to EDL formation in supercapacitors, without extending the scope to detailed ion–ion energy partitioning. Results for the *E*_C_ and *E*_LJ_ energies per ionic pairs between the electrolyte species and the electrodes for the systems submitted of a surface density charge of ±4.81 μC/cm^2^ are presented in Figures 5 and 6, respectively. The evaluation of *E*_C_ between electrolyte species and electrodes provides microscopic insight into the role of each ion or molecule in shaping the interfacial layers. In general, the magnitude of these interactions increases with higher surface charge density, reflecting stronger local polarization and enhanced electrostatic coupling at the interface. At a surface charge density of σ = ±4.81 μC/cm^2^, the [bmim] cation exhibits strong attraction to the negative electrode across all models, with values ranging from −0.76 to −0.86 kcal/mol. This consistent behavior confirms the dominant role of [bmim] in structuring the negative EDL, where it preferentially accumulates at the negatively charged graphene surface. Conversely, its interaction with the positive electrode remains weakly repulsive, with values between +0.17 and +0.26 kcal/mol.

For the anions, significant differences are observed among the systems. In the [ace]- and [prop]-based electrolytes (M_A_ and M_C_), interactions with the positive electrode reach −0.27 kcal/mol ([ace]) and −0.24 kcal/mol ([prop]) at most, indicating a relatively limited contribution to the structuring of the positive EDL. In contrast, in M_B_ ([bmim]­[bnz]), the [bnz] anion shows a substantially stronger interaction (−0.44 kcal/mol), emerging as the anion with the highest affinity for the positive interface.

In the mixed systems, [bnz] remains the key player. In M_AB_ ([bmim]­[ace] + [bmim]­[bnz]), its interaction with the positive electrode (−0.31 kcal/mol) clearly surpasses that of [ace] (−0.07 kcal/mol). Similarly, in M_BC_ ([bmim]­[bnz] + [bmim]­[prop]), [bnz] reaches −0.32 kcal/mol, while [prop] remains much weaker (−0.05 kcal/mol). Even in the ternary M_ABC_ system, [bnz] continues to dominate (−0.26 kcal/mol), whereas [ace] and [prop] contribute negligibly (−0.03 to −0.06 kcal/mol). Therefore, at σ = ±4.81 μC/cm^2^ a clear pattern emerges: [bmim] governs the negative EDL in all systems, while [bnz] dominates the positive EDL whenever present in the electrolyte, with interaction energies up to three times stronger than those of [ace] and [prop]. These findings suggest that electrolytes containing [bnz] yield more stable and better-structured EDLs, which may translate into enhanced differential capacitance.

On the other hand, at the highest surface charge density (σ = ±4.81 μC/cm^2^), *E*_LJ_ highlight the key role of van der Waals forces in shaping the EDLs. Overall, the [bmim] cation exhibits the strongest dispersive interactions with the negative electrode, reaching −5.8 kcal/mol across all systems. This strong LJ contribution complements the Coulomb attraction already observed, confirming [bmim] as the dominant species structuring the negative EDL. With the positive electrode, [bmim]–Graphene+ interactions are much weaker (−1.5 to −2.2 kcal/mol), consistent with its low affinity for this interface.

Anions display distinct behaviors. Both [ace] and [prop] ions show moderate interactions with the positive electrode (−0.4 kcal/mol in mixed models to −1.7 kcal/mol in pure hydrated models), whereas [bnz] clearly stands out with stronger values, up to −2.7 kcal/mol in the M_BC_ system and −2.1 kcal/mol in M_ABC_. This quantitative difference highlights [bnz]’s higher propensity for adsorption at the positive graphene surface. Water also plays a significant role: dispersive interactions range from −3.0 to −3.7 kcal/mol with the positive electrode, and from −1.2 to −1.8 kcal/mol with the negative electrode. These values reinforce the high mobility and infiltration capacity of water, consistent with density profile observations.

In mixed systems, [bnz] again dominates. In M_AB_ ([bmim]­[ace] + [bmim]­[bnz]), [bnz]–Graphene+ (−2.7 kcal/mol) is clearly stronger than [ace]–Graphene+ (−0.34 kcal/mol). In M_BC_ ([bmim]­[bnz] + [bmim]­[prop]), [bnz] reaches −2.76 kcal/mol, while [prop] remains weak (−0.43 kcal/mol). Even in the ternary M_ABC_ system, [bnz] maintains the strongest LJ interaction with the positive electrode (−2.16 kcal/mol), whereas [ace] and [prop] remain negligible (−0.16 and −0.44 kcal/mol, respectively). Therefore, at σ = ±4.81 μC/cm^2^, Lennard-Jones interactions reinforce the same trends identified from Coulomb energies: [bmim] dominates the negative EDL, while [bnz] governs the positive EDL with up to three times stronger interactions compared to [ace] and [prop]. These results suggest that [bnz] is the most effective anion in promoting stable adsorption at the positive interface, leading to a more defined EDL structure and potentially higher differential capacitance. Although classical MD does not treat π–π or cation−π interactions explicitly, the force-field parameters assigned to aromatic rings and to the graphene surface effectively incorporate these contributions through calibrated partial charges and Lennard–Jones terms. As a result, the benzoate anion may experience enhanced stabilization near the carbon electrode due to stronger effective dispersion and electrostatic interactions compared to the more hydrophilic acetate and propionate anions. This effective aromatic–surface affinity acts in combination with hydration structure and electrostatic screening to modulate the interfacial ion distributions observed in our simulations.

### 3.2 Electrical Properties Analysis

By applying the one-dimensional Poisson equation, the electrostatic potential profiles of all devices were obtained along the *z*-axis, and the potential differences corrected by the point of zero charge (PZC) were subsequently determined. The corresponding values are reported in Table 3. It is important to stress that the absolute potential values should not be directly compared across different models, since the reference level is arbitrary and depends on boundary conditions and the internal alignment of the potential within the simulation box. For this reason, the most meaningful quantity is the PZC-corrected potential difference (ΔΦ), which removes the arbitrariness of the zero-point and more faithfully reflects the electrostatic response of the systems under polarization. At zero surface charge, all models display ΔΦ values close to zero, with variations below 0.03 V. This confirms the consistency of the PZC correction and indicates that the systems where the electrodes are in a zero-charge condition, there are small interactions between the molecules and the graphenes that result in a small residual potential difference. As the surface charge density increases, ΔΦ grows almost linearly for all devices, reflecting the progressive increase of the potential drop between the electrodes as the EDLs reorganize under stronger electric fields.

**Table 3:** Electrostatic Potential Values at the Positive and Negative Electrodes (Φ^+^ and Φ^–^, Respectively), Together with the Total Potential Difference Corrected by the PZC (ΔΦ), for All Surface Charge Densities Investigated in This Study

model	σ (μC/cm^2^)	Φ^–^ (V)	Φ^–^ (V)	ΔΦ (V)
M_A_	0.00	0.7975	0.7840	0.0136
	1.60	1.0294	0.4074	0.6085
	3.20	1.2862	0.0006	1.2721
	4.81	1.5066	–0.4146	1.9077
M_B_	0.00	0.7298	0.7301	–0.0003
	1.60	0.9622	0.3767	0.5858
	3.20	1.1440	–0.0045	1.1489
	4.81	1.3583	–0.4611	1.8197
M_C_	0.00	0.7804	0.7752	–0.0272
	1.60	1.0372	0.3859	0.7549
	3.20	1.2692	–0.0198	1.7549
	4.81	1.5129	–0.4422	2.1086
M_AB_	0.00	0.7363	0.7353	0.0010
	1.60	0.9852	0.4436	0.5406
	3.20	1.2212	0.1139	1.1063
	4.81	1.4596	–0.2389	1.6976
M_AC_	0.00	0.7615	0.7487	0.0128
	1.60	1.0521	0.4452	0.5941
	3.20	1.3201	0.1210	1.1862
	4.81	1.5744	–0.2421	1.8036
M_BC_	0.00	0.7289	0.7465	–0.0176
	1.60	0.9977	0.4387	0.5765
	3.20	1.2344	0.1176	1.1344
	4.81	1.4765	–0.2515	1.7456
M_ABC_	0.00	0.7458	0.7278	0.0181
	1.60	0.9989	0.4471	0.5389
	3.20	1.2581	0.1122	1.1331
	4.81	1.4962	–0.2447	1.7281

At σ = ±1.60 μC/cm^2^, ΔΦ values range from 0.54 to 0.75 V, with the highest observed for model M_C_ ([bmim]­[prop]), while mixed systems containing [bnz] (M_AB_ and M_ABC_) exhibit the lowest values. At σ = ±3.20 μC/cm^2^, the separation among models becomes more evident, with ΔΦ spanning from ∼1.10 to 1.75 V. Once again, M_C_ shows the largest potential drop, indicating that it requires a stronger electrostatic difference to accumulate the same surface charge density, which translates into a lower differential capacitance. In contrast, M_AB_, M_ABC_, and M_BC_ display the smallest ΔΦ values, suggesting higher capacitance. At the highest polarization studied, σ = ±4.81 μC/cm^2^, the same trend is maintained: M_C_ reaches the largest ΔΦ (2.11 V), whereas M_AB_ exhibits the lowest (1.70 V), closely followed by M_ABC_ and M_BC_. All systems exhibit an approximately linear increase of ΔΦ with σ, marked differences are observed in their electrostatic response. The [prop]-based M_C_ model consistently appears as the least efficient, presenting the highest ΔΦ across all charge densities, while the [bnz]-containing systems, particularly M_AB_, M_BC_, and M_ABC_, show a more favorable response, characterized by lower potential differences and, consequently, higher differential capacitance.

To confirm the trends observed in the analysis of the total potential difference corrected by the PZC, we investigated the charge distribution in the regions adjacent to the electrodes. A 0.5 nm region was selected from each graphene surface and subdivided into three sublayers, denoted Q1, Q2, and Q3, based on the position of the characteristic water density peaks in the mass density profiles, which provide a natural criterion for segmenting the EDL structure. The charge density was then integrated in each sublayer to obtain the net charge, according to $Q_{k}=\int_{Z_{k}}^{z_{k+1}}\rho_{q}\left(z\right)dz$ , where *k* = 1, 2, and 3; ρ_*q*_(*z*) is the laterally averaged charge density along the *z*-axis, and the integration limits *z*_*k*_ and *z*_*k*+1_ correspond to the water-defined sublayer boundaries. This methodology enables a spatially resolved description of how different ionic and molecular species contribute to the neutralization of the electrode charges. The results for the positive and negative EDLs, considering 0.5 nm from the electrode surface, are presented in Figures 7 and 8, respectively.

**Figure 7:**
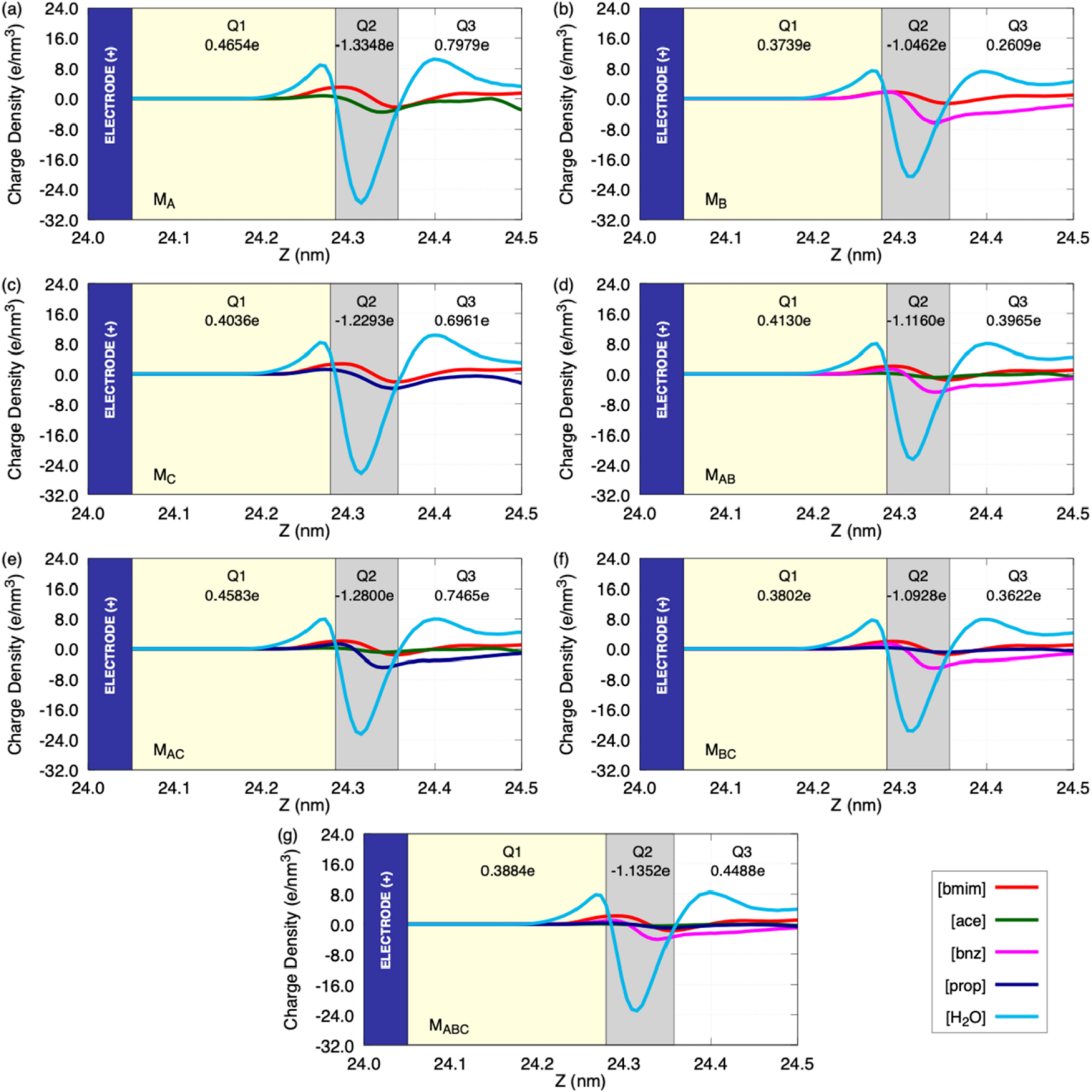
Integrated net charge in the positive EDL (EDL+), considering 0.5 nm from the electrode surface. The EDL was partitioned into three sublayers (Q_1_, Q_2_, and Q_3_) according to the position of the water density peaks, and the net charge in each region was obtained by integrating the charge density profile. The models are indicated as follows: (a) M_A_; (b) M_B_; (c) M_C_; (d) M_AB_; (e) M_AC_; (f) M_BC_; and (g) M_ABC_. The blue bar represents the positive electrode.

**Figure 8:**
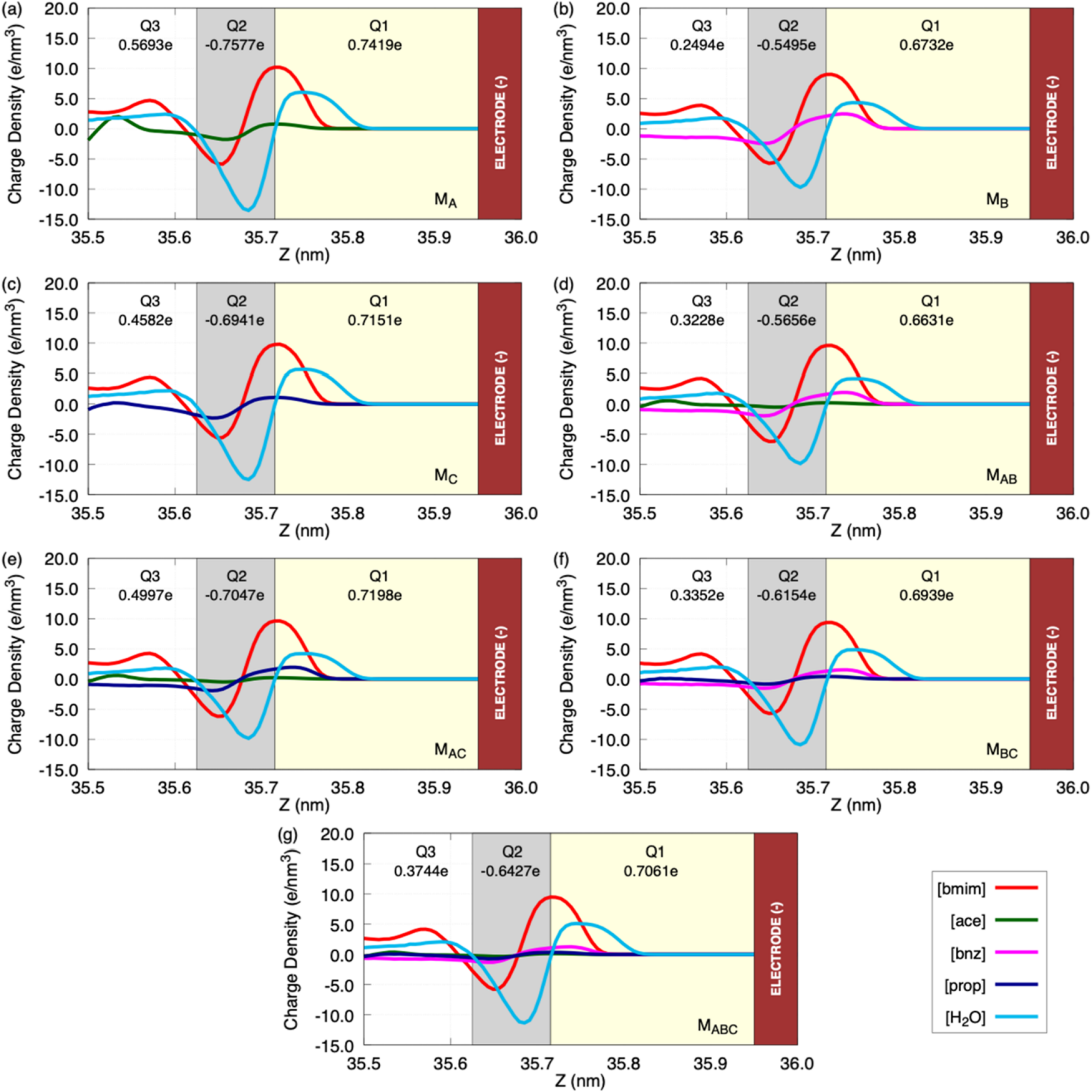
Integrated net charge in the negative EDL (EDL−), considering 0.5 nm from the electrode surface. The EDL was partitioned into three sublayers (Q_1_, Q_2_, and Q_3_) according to the position of the water density peaks, and the net charge in each region was obtained by integrating the charge density profile. The models are indicated as follows: (a) M_A_; (b) M_B_; (c) M_C_; (d) M_AB_; (e) M_AC_; (f) M_BC_; and (g) M_ABC_. The red bar represents the negative electrode.

With the partitioning of the EDL into *Q*_1_ (contact), *Q*_2_ (first layer), and *Q*_3_ (second layer), within 0.5 nm from the positive graphene surface, it is possible to confirm the alternation of electric charge by region and the behavior of the total charge *Q* = *Q*_1_ + *Q*_2_ + *Q*_3_. In the vicinity of the positive graphene electrode, the pattern *Q*_1_ > 0, *Q*_2_ < 0, and typically *Q*_3_ ≥ 0 results in a net negative charge (*Q*) for all models, but with very different magnitudes: *Q*(*M*_A_) ≈ −0.052e, *Q*(*M*_AC_) ≈ −0.075e, *Q*(*M*_C_) ≈ −0.130e, *Q*(*M*_AB_) ≈ −0.307e, *Q*(*M*_ABC_) ≈ −0.298e, *Q*(*M*_BC_) ≈ −0.350e, and *Q*(*M*_B_) ≈ −0.411e. Thus, M_B_, M_BC_, M_ABC_, and M_AB_ provide a stronger countercharge at the positive electrode interface, while M_A_, M_AC_, and M_C_ (especially M_A_) are clearly weaker. This shows that, for several systems, the bottleneck of the electrostatic response may lie on the positive side when the countercharge *Q* is small. In the vicinity of the negative electrode, the signs are reversed (*Q*_1_ > 0, *Q*_2_ < 0, and *Q*_3_ > 0), and the net charge is positive for all models: *Q*(*M*_B_) ≈ +0.373e, *Q*(*M*_BC_) ≈ +0.414e, *Q*(*M*_AB_) ≈ +0.420e, *Q*(*M*_ABC_) ≈ +0.438e, *Q*(*M*_C_) ≈ +0.479e, *Q*(*M*_AC_) ≈ +0.515e, and *Q*(*M*_A_) ≈ +0.554e. Here, M_B_ provides the lowest net charge, while M_A_ and M_AC_ provide the strongest values. Considering the potential differences (ΔΦ) observed earlier: M_C_ shows a weak countercharge at positive graphene and therefore exhibits the largest ΔΦ; M_AB_, M_BC_, and M_ABC_ sustain stronger countercharges at both electrodes, resulting in smaller ΔΦ; M_A_ and M_AC_ are limited by a weak countercharge near the positive electrode despite being strong at the negative one; and M_B_ combines a very strong electrolyte charge distribution near the positive electrode with a very weak one near the negative electrode, which translates into an intermediate ΔΦ. Minor deviations are still expected, since capacitance depends not only on the amount of countercharge but also on the local electrolyte structuring.

To quantitatively determine the specific capacitance of each electrode and, consequently, the overall capacitance of the devices, a linear fit of the form φ­(*x*) = α*x* + β was performed. This procedure, applied to the σ × Φ representation, allowed extracting the slopes associated with the differential capacitances. The results of this fitting for all investigated models are shown in Figure 9, while the values of the specific capacitance of the electrodes and the total capacitance is highlighted in Table 4. The capacitance values reported in Table 4 reinforce the trends previously discussed from the analysis of potential differences (ΔΦ) and the integrated charge distribution in the *Q*_1_ – *Q*_3_ sublayers of the EDLs. In general, the values of *C* ^+^ are relatively similar across systems (≈ 6.0–7.7 μF/cm^2^), and *C* ^*–*^ ≈ 4.0–4.9 μF/cm^2^. As a result, the total capacitance *C* ^Tot^, derived from the series connection of the two electrodes, is predominantly limited by the performance of the negative electrode. Among the systems, M_AB_ exhibits the highest *C* ^Tot^ (2.83 μF/cm^2^), followed by M_BC_ and M_ABC_ (both 2.76 μF/cm^2^). This result is consistent with the previous observations: mixtures containing [bnz] displayed lower ΔΦ values and highest *Q* countercharges at both electrodes’ vicinity. In contrast, M_A_ and M_C_, which showed weaker *Q* countercharge at positive electrode and the largest ΔΦ, present the lowest total capacitances (2.51 and 2.47 μF/cm^2^, respectively). M_B_, despite containing [bnz], yields an intermediate *C* ^Tot^ (2.66 μF/cm^2^), reflecting the imbalance previously noted between its strong compensation at positive graphene’ EDL and the weakest at negative graphene’ EDL. Similarly, M_AC_ shows an intermediate value (2.67 μF/cm^2^), consistent with the more moderate compensation found in the countercharge analysis. Overall, the capacitance analysis quantitatively confirms the earlier conclusions: the presence of [bnz], particularly in mixed systems, plays a key role in enhancing total capacitance, while systems based solely on [ace] or [prop] are comparatively less efficient.

**Figure 9:**
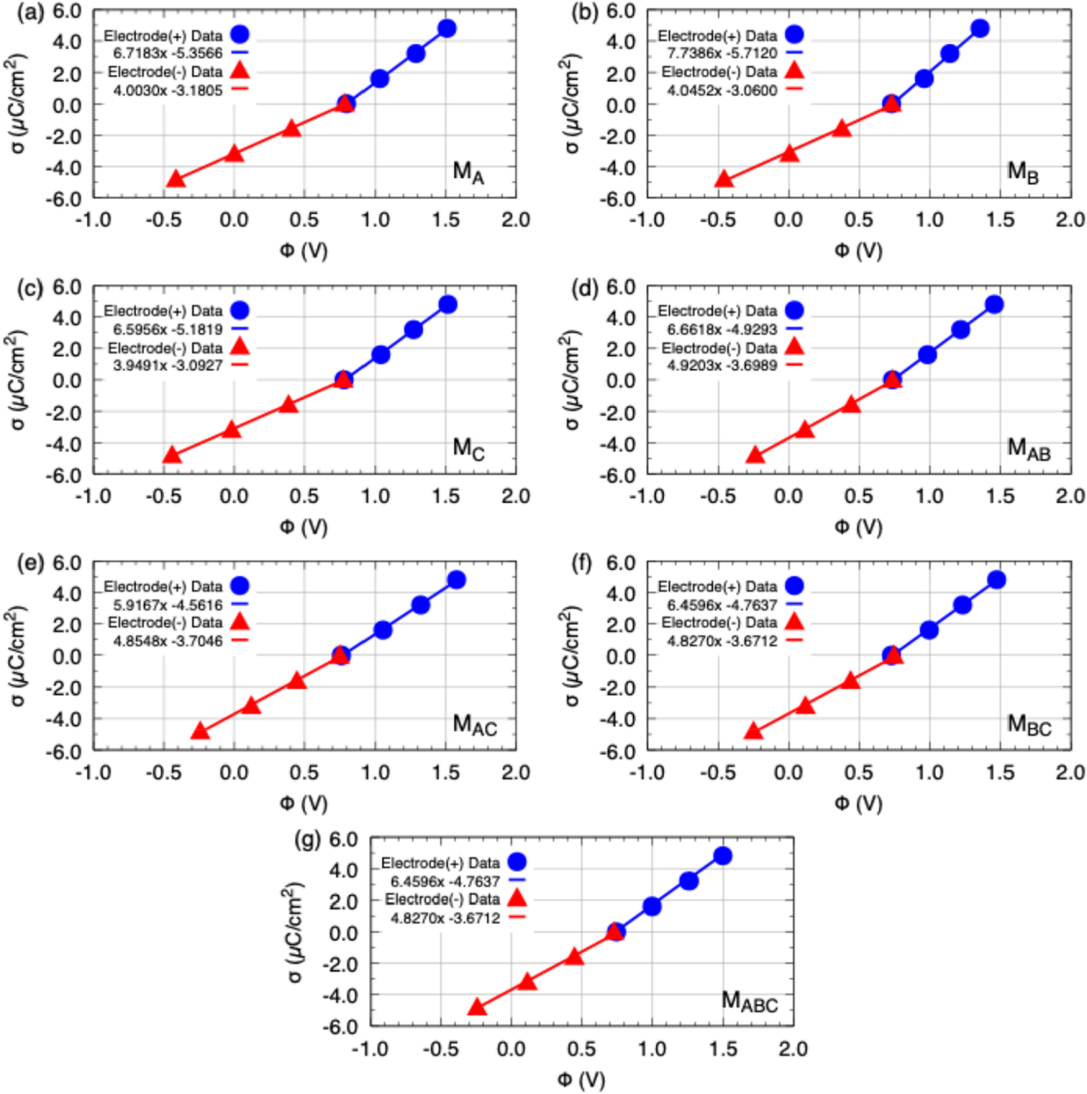
σ × Φ representation together with the linear fit in the form ϕ­(*x*) = α*x* + β for the models: (a) M_A_, (b) M_B_, (c) M_C_, (d) M_AB_, (e) M_AC_, (f) M_BC_, and (g) M_ABC_. The corresponding equation is displayed in the key of each panel. The slope of the line represents the specific capacitance of the electrodes.

**Table 4:** Values of Capacitances in the Positive Electrode (*C* ^+^), Negative Electrode (*C* ^–^) and Total Capacitance (*C* ^Tot^) Considering the Series Connection between the Electrodes

model	*C* ^+^ (μF/cm^2^)	*C* ^–^ (μF/cm^2^)	*C* ^Tot^ (μF/cm^2^)
M_A_	6.72	4.00	2.51
M_B_	7.74	4.05	2.66
M_C_	6.60	3.95	2.47
M_AB_	6.66	4.92	2.83
M_AC_	5.92	4.85	2.67
M_BC_	6.46	4.83	2.76
M_ABC_	6.46	4.83	2.76

From the total capacitance values and the potential differences, the amount of energy stored per unit mass and per unit volume was calculated, corresponding to the gravimetric and volumetric energy densities, respectively, according to the equations provided. The results are summarized in Table 5. The gravimetric and volumetric energy densities follow the expected trend of increasing with surface charge density, reflecting the greater energy stored in the devices under stronger polarization. At σ = ±4.81 μC/cm^2^, M_C_ reaches 4.06 J/g and 4.58 J/cm^3^, while the others remain within 2.97–3.36 J/g and 3.40–3.81 J/cm^3^. These results highlight an apparent discrepancy compared to the capacitance and ΔΦ analyses. While M_C_ was previously identified as the least efficient in terms of differential capacitance (largest ΔΦ and lowest *C* ^Tot^), here it displays the highest energy densities. This difference arises because energy depends not only on capacitance but also on the square of the potential difference $\left(U=\frac{1}{2}C^{\mathrm{Tot}}{\left(\delta \delta \Phi \right)}^{2}\right)$ . Thus, despite its lower capacitance, the higher ΔΦ achieved in M_C_ compensates for this limitation, yielding superior gravimetric and volumetric energy densities.

**Table 5:** Values of Gravimetric Energy Density and Volumetric Energy Density for All Models Studied at σ = ±4.81 μC/cm^2^

model	*u*_*m*_ (J/g)	*u*_*v*_ (J/cm^3^)
M_A_	3.36	3.81
M_B_	3.19	3.67
M_C_	4.06	4.58
M_AB_	2.97	3.40
M_AC_	3.20	3.62
M_BC_	3.07	3.50
M_ABC_	3.01	3.43

Conversely, the [bnz]-containing models (M_AB_, M_BC_, and M_ABC_), which showed higher total capacitances and lower ΔΦ, display intermediate energy densities, suggesting they are more efficient electrostatically but store less energy compared to M_C_. Models M_A_, M_B_, and M_AC_ exhibit similar intermediate behavior, without significant distinction, reflecting a balance between capacitance and electric potential. It is important to emphasize that the direct comparison of energy densities among the different models, as presented so far, may be considered unfair, since each system operates under distinct potential differences. To reliably assess which device truly exhibits superior electrochemical performance, all systems must be compared under the same potential difference conditions. For this purpose, a fit of the form Ψ­(*x*) = γ*x* ^2^ was performed, allowing the potential difference ΔΦ to be fixed and the energy storage capability of the systems to be consistently compared. The graphical representation of this fitting for both gravimetric and volumetric energy densities is shown in Figure 10. The values for *u*_*m*_ and *u*_*v*_ at a fixed potential difference of 2.5 V are shown in Table 6.

**Figure 10:**
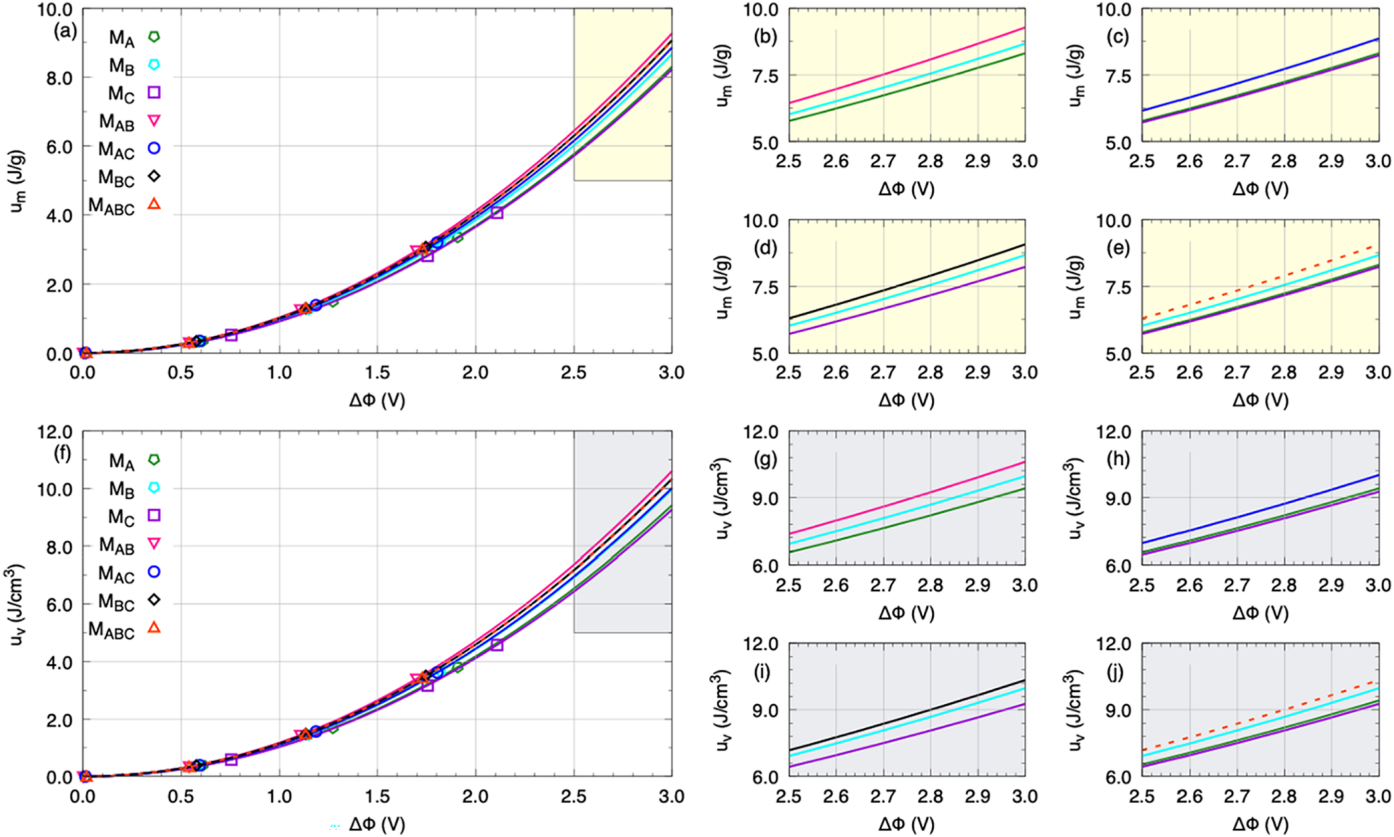
Gravimetric (*u*_*m*_) (panels a–e) and volumetric (*u*_*v*_) (panels f–j) energy densities for all models. Panels (b–d) highlight, in yellow, the 2.0–2.5 V region for *u*_*m*_, distributed as follows: (b) M_A_, M_B_, and M_AB_; (c) M_A_, M_C_, and M_AC_; (d) M_B_, M_C_, and M_BC_; and (e) M_A_, M_B_, M_C_, and M_ABC_. Panels (g–j) shows the same region (2.0–2.5 V) for *u*_*v*_, highlighted in gray, organized as (g) M_A_, M_B_, and M_AB_; (h) M_A_, M_C_, and M_AC_; (i) M_B_, M_C_, and M_BC_; and (j) M_A_, M_B_, M_C_, and M_ABC_.

**Table 6:** Values of Gravimetric Energy Density and Volumetric Energy Density at a Fixed Potential Difference of 2.5 V for All Models Studied

model	*u*_*m*_ (J/g)	*u*_*v*_ (J/cm^3^)
M_A_	5.77	6.54
M_B_	6.02	6.93
M_C_	5.71	6.44
M_AB_	6.44	7.37
M_AC_	6.15	6.96
M_BC_	6.30	7.18
M_ABC_	6.30	7.18

The gravimetric and volumetric energy density values obtained at a fixed potential difference of 2.5 V provide a fair comparison among the systems, eliminating the bias associated with the distinct potential drops originally observed for each electrolyte. Under these conditions, the models exhibit performance with ranging from 5.7 to 6.4 J/g (6.4 and 7.4 J/cm^3^). Among the pure electrolytes with water, M_B_ stands out with the highest performance (6.02 J/g and 6.93 J/cm^3^), while M_C_ shows the lowest values (5.71 J/g and 6.44 J/cm^3^). M_A_ lies in between but closer to M_C_. The mixtures, however, show a clear enhancement: M_AB_ achieves the highest values overall (6.44 J/g and 7.37 J/cm^3^), followed by M_BC_ and the ternary mixture M_ABC_, both with nearly identical performance (≈6.30 J/g and 7.18 J/cm^3^). These results reinforce the role of the [bnz] anion in enhancing energy storage capability, in agreement with previous analyses of capacitance and ΔΦ. Therefore, the analysis demonstrates that the best electrochemical performance under fixed-potential conditions is obtained with electrolytes containing [bnz], either pure or in mixtures, with the [ace] + [bnz] combination delivering the highest gravimetric and volumetric energy densities.

## 4 Conclusions

In this work, we carried out a detailed molecular dynamics investigation of graphene-based supercapacitors employing hydrated ester-derived ionic liquids, examining their behavior both in pure form and as binary and ternary mixtures. The constant-charge framework adopted here ensured consistent interfacial conditions across all systems and enabled a direct evaluation of how ester-based anions influence the structure and electrochemical response of the electric double layer (EDL). Our results demonstrate that all electrolytes form well-defined EDLs exhibiting alternating charge regions, moderate overscreening, and a nearly linear dependence of the PZC-corrected potential drop on surface charge density. Among the investigated anions, benzoate consistently showed the strongest affinity for the graphene surface, leading to more compact and symmetric EDLs and yielding the highest differential capacitances, whereas acetate and propanoate displayed weaker interfacial screening. Energy-storage analysis further revealed that although the propanoate-based electrolyte reaches the highest absolute gravimetric and volumetric energy densities due to its larger potential difference, mixtures containing benzoate deliver the best normalized performance when all systems are compared under a fixed operating voltage, highlighting their superior charge-storage efficiency.

Beyond the quantitative findings, this study introduces new conceptual insights into how ester-derived anions can be used as molecular design elements to tune interfacial organization in hydrated ionic-liquid electrolytes. The results clarify that the enhanced performance of benzoate-containing systems arises from a combination of favorable dispersion interactions with the carbon surface, reduced hydration affinity, and more effective competition for inner-layer adsorption under confinement. These mechanistic details deepen the understanding of hydration-controlled EDL restructuring and complement previous studies showing that electrolyte hydration can enhance the electrochemical response of ionic-liquid-based supercapacitors. The present results further advance the field by demonstrating that the negative electrode is the main limiting interface for total capacitance in these systems and by providing a microscopic rationale for the interplay between electrostatic, dispersive, and hydrogen-bonding interactions in shaping interfacial charge compensation.

Overall, the findings of this work establish hydrated ester-derived ionic liquids as a promising platform for the rational design of sustainable and high-performance electrolytes. They emphasize that the molecular characteristics of the anion—notably size, aromaticity, and hydration behavior—play a decisive role in tuning the efficiency of graphene-based supercapacitors. These results also suggest several directions for future research, including the exploration of other aromatic ester-derived anions, the integration of constant-potential simulations to refine the description of electrode polarization, and the investigation of temperature effects or alternative cosolvents to optimize the balance between mobility and capacitance. In addition, coupling the present insights with data-driven or machine-learning strategies may accelerate the targeted development of next-generation green electrolyte formulations. Collectively, this study provides a coherent microscopic foundation for understanding and improving ester-based ionic-liquid electrolytes in carbon-based energy-storage devices, and it lays the groundwork for future advances in the design of more efficient, sustainable supercapacitors.

## Supplementary Material



## Notes

Complete mathematical derivation of the equations used to calculate the number of ionic liquid ion pairs and water molecules in electrolyte systems at a fixed concentration of 2 M, including formulations for systems containing one, two, and three ionic liquids and the assumptions of volume additivity (PDF)
