# Supplementary material for: Ester-Based Electrolyte Mixtures for Graphene Supercapacitors: A Molecular Dynamics Investigation
Source: J Phys Chem B. 2025 Dec 23;130(7):2178–93. doi: 10.1021/acs.jpcb.5c06507 (PMC12926949; doi:10.1021/acs.jpcb.5c06507)
Supplement: Supplementary file 1 [file jp5c06507_si_001.pdf]

S U P P O R T   I N F O R M A T I O N

Ester-Based Electrolyte Mixtures for Graphene Supercapacitors: A Molecular Dynamics Investigation

Lucas de S. Silva[1] and Guilherme Colherinhas[1*]

1. *Instituto de Física. Universidade Federal de Goiás. 74690-900. Goiânia. GO. Brazil.*

Derivation of Molecular Count Equations for Ionic Liquid + Water Systems

Here we present the full mathematical derivation of the equations used to compute the number of ionic liquid (IL) molecules and water molecules for systems constructed at a fixed electrolyte concentration of 2 M. Derivations are provided for systems containing one, two, and three ionic liquids plus water.

- General Definitions:

Let:

$-N_i$ = number of molecules (ion pairs) of ionic liquid i

$-n_i = \frac{N_i}{N_A}$ = moles of ionic liquid i

- N_A = Avogadro's number
- ρ_i = density of ionic liquid i
- M_i = molar mass of ionic liquid i
- C = concentration (2 M in this study)
- V_{box} = volume of simulation box
- V_{H_2O} = volume of water
- N_{H_2O} = number of water molecules

We assume approximate volume additivity:

$$V_{box} = \sum \left(n_i \frac{M_i}{\rho_i} \right) + V_{H_2O}$$

<u>Case 1: One Ionic Liquid + Water:</u>

Concentration definition:

$$C = \frac{n}{V_{H_2O}} \rightarrow n = C\, V_{H_2O}$$

Volume relation:

$$V_{box} = \left(n \frac{M}{\rho} \right) + V_{H_2O}$$

Substitute n:

$$V_{box} = \left(\frac{CV_{H_2O}M}{\rho}\right) + V_{H_2O}$$

$$V_{box} = V_{H_2O}\left(1 + \frac{CM}{\rho}\right)$$

Solve for V_{H_2O}:

$$V_{H_2O} = \frac{V_{box}\rho}{\rho + CM}$$

Then $n = CV_{H_2O}$, so:

$$n = \frac{CV_{box}\rho}{\rho + CM}$$

Convert to molecules:

$$N = nN_A = \left(CV_{box}\frac{\rho}{\rho + CM}\right)N_A$$

Water molecules:

$$N_{H_2O} = \frac{N\rho_{H_2O}}{CM_{H_2O}}$$

<u>Case 2: Two Ionic Liquids + Water:</u>

$$C = \frac{n_1 + n_2}{V_{H_2O}} \rightarrow n_1 = CV_{H_2O} - n_2$$

Volume relation:

$$V_{box} = (\frac{n_1M_1}{\rho_1}) + (\frac{n_2M_2}{\rho_2}) + V_{H_2O}$$

Substitute n_1:

$$V_{box} = \left(\frac{\left(CV_{H_2O} - n_2\right)M_1}{\rho_1}\right) + \left(\frac{n_2M_2}{\rho_2}\right) + V_{H_2O}$$

Solve for V_{H_2O} and substitute back to find n_1. Final expression it will be:

$$N_1 = \frac{N_A\rho_1}{\rho_1 + CM_1}[CV_{box} - \left(\frac{N_2}{N_A}\right)\left(1\ + \frac{CM_2}{\rho_2}\right)]$$

Water molecules:

$$N_{H_2O} = \frac{(N_1 + N_2)\rho_{h_2o}}{CM_{H_2O}}$$

Case 3: Three Ionic Liquids + Water:

$$C = \frac{n_1 + n_2 + n_3}{V_{H_2O}} \rightarrow n_1 = CV_{H_2O} - n_2 - n_3$$

Volume relation:

$$V_{box} = \left(\frac{n_1M_1}{\rho_1}\right) + \left(\frac{n_2M_2}{\rho_2}\right) + \left(\frac{n_3M_3}{\rho_3}\right) + V_{H_2O}$$

Substitute n_1, solve for V_{H_2O}, substitute back. Final expression will be:

$$N_1 = \frac{N_A\rho_1}{\rho_1 + CM_1}[CV_{box} - \left(\frac{N_2}{N_A}\right)\left(1 + \frac{CM_2}{\rho_2}\right) - \left(\frac{N_3}{N_A}\right)\left(1 + \frac{CM_3}{\rho_3}\right)]$$

Water molecules:

$$N_{H_2O} = \frac{(N_1 + N_2 + N_3)\rho_{H_2O}}{CM_{H_2O}}$$

Remarks

• No molecule number is arbitrary; all systems maintain C = 2 M exactly.

• Equations ensure thermodynamic consistency.

• The method generalizes to any number of ionic species.

• These formulas ensure fair comparison between pure and mixed electrolytes.
